# Association between obesity, sex, medical comorbidities, and survival in cancer patients treated with immune checkpoint inhibitors

**DOI:** 10.7150/jca.130032

**Published:** 2026-06-10

**Authors:** Mingjia Li, Daniel J. Spakowicz, Songzhu Zhao, Hyunwoo Kwon, Kenneth Chian, Adam Khorasanchi, Yizhen Guo, Lai Wei, Lingbin Meng, Yuanquan Yang, Asrar Alahmadi, Regan M. Memmott, Jacob Kaufman, Kai He, Peter G. Shields, Thomas A. Mace, Mitch A. Phelps, Christopher C. Coss, Zihai Li, Kari L. Kendra, David P. Carbone, Gregory A. Otterson, Carolyn J. Presley, Dwight H. Owen

**Affiliations:** 1 Division of Medical Oncology, Department of Internal Medicine, The Ohio State University, Columbus, USA.; 2 Pelotonia Institute for Immuno-Oncology, The Ohio State University, Columbus, USA.; 3 Department of Biomedical Informatics, Center for Biostatistics, The Ohio State University, Columbus, USA.; 4 Department of Internal Medicine, The Ohio State University, Columbus, USA.; 5 College of Medicine Medical Scientist Training Program, The Ohio State University, Columbus, USA.; 6 Division of Gastroenterology Hepatology and Nutrition, Department of Internal Medicine, The Ohio State University, Columbus, USA.; 7 College of Pharmacy, The Ohio State University, Columbus, USA.; 8 College of Medicine, The Ohio State University, Columbus, USA.

## Abstract

**Background:**

Obesity's impact on cancer treatment outcomes is poorly understood, especially in the context of immuno-oncology. This study explores how obesity and medical comorbidities are associated with overall survival in cancer patients receiving immune checkpoint inhibitors (ICIs). Additionally, considering the influence of sex on body composition in obesity, this study examines the relationship between sex, obesity, medical comorbidities, and survival.

**Methods:**

This cohort study involved 688 patients with metastatic cancer received ICIs as first- or second-line therapy. Obesity was assessed using body mass index (BMI). Cox proportional hazard models and Kaplan-Meier survival analysis were used to examine associations between predictors and overall survival.

**Results:**

Patients with higher BMI had longer overall survival, and hazard ratio (HR) for death was 0.83 (95% CI 0.73-0.95) for every 10 units increased in BMI. Additionally, patients belonged to the highest BMI group (≥ 40) had the lowest risk of death when comparing to patients with BMI 18.5 to < 30 with HR 0.58 (95% CI 0.37-0.90). In subgroup analysis, a significant association between high BMI and decreased HR for death was predominantly observed in the male cohort. In multivariate analysis, the prognostic value of BMI remained significant after adjusting for performance status, line of therapy, age-adjusted medical comorbidities, and cancer type.

**Conclusions:**

Obesity was associated with decreased mortality risk for cancer patients who had received ICIs. There could be a sex-dependent association between survival benefit and obesity.

## 1. Introduction

Obesity is one of the most prevalent comorbidities 1. According to the World Health Organization (WHO), 13% of people aged 18 or greater are obese worldwide 2. In the United States, the Center for Disease Control estimated 42.4% of adults were obese 3. Obesity has important implications in cancer care given its pivotal role in oncogenesis 4, 5. Conservatively, 10-20% of cancers are contributed to obesity 6, 7.

The effects of obesity on health are complex. Obesity has profound influences on anti-cancer immunity, and has been linked with higher risk of developing cancer due to dysregulation on immune surveillance 8. Earlier studies have demonstrated increased inflammatory cytokines such as tumor necrosis factor α, interferon γ, interleukin (IL) -1, and IL-6 in obese cancer patients 9, 10. Aberrant production, distribution, and function of natural killer (NK) cells, T-lymphocytes, and antigen-presenting cells have been associated with obesity 11. Furthermore, multiple studies have demonstrated upregulations in signal transducer and activator of transcription 3 (STAT3) and programmed cell death protein 1 (PD-1) in obesity, which is thought to occur via increased leptin levels 12-14. In addition to altered immunity, obesity reflects underlying nutritional status and influences treatment outcome by affecting drug metabolism 15-17.

Prior to the wide adaptations of immune checkpoint inhibitors (ICIs), obesity was associated with increased mortality in all cancers 18, 19. This negative association has been revisited in the era of ICI-based treatments. Multiple retrospective studies have reported higher response rates and longer survival in obese cancer patients treated with ICIs 20-23. In addition, this observed “obesity paradox” was more profound in male population 24, 25. Although the exact mechanisms pertaining to obesity paradox have remained unclear, one potential mechanism proposes is the exhausted PD-1+ CD8+ T cells within obese immunosuppressive milieu have been more responsive to ICI. Furthermore, androgen-driven mechanism of CD8+ T cell exhaustion and improved PD-1 targeted therapy upon its blockade may contribute to increased ICI efficacy in obese males with marked alterations in androgen metabolism 26-30.

In this study, we investigated the relationships between obesity, sex, and clinical outcomes for patients with metastatic cancer treated with ICIs.

## 2. Methods

### 2.1 Study Design and Sample

We conducted a retrospective cohort study of 688 patients with metastatic cancer treated with immune checkpoint inhibitors. This study was reviewed and approved by the institutional review board (IRB 2016C0070). All patients received at least 1 dose of an immune checkpoint inhibitor as either first- or second-line therapy between 2011 and 2018 and remained under follow up until July 2020.

Clinical variables such as patients' demographics, tumor characteristics, and survival outcomes were collected via chart review. BMI was collected at baseline prior to the start of ICIs. BMI was defined as weight in kilograms divided by square height in meters (BMI = kg/m2). Charlson Comorbidity Index (CCI) was abstracted through query of ICD-10 codes for each patient 31. Comorbidity data was manually extracted via chart review for 13 out of 688 patients in the all-cancer cohort who had missing ICD-10 data. A Modified CCI score excluding points assigned for cancer diagnosis was used for this study (Supplemental Table 1). Obesity was defined according to the WHO classification, which categorizes BMI ranges to determine obesity levels: obesity (class 1 30.0-34.9 and class 2 35.0-39.9), and extreme obesity (class 3) ≥ 40.

### 2.2 Statistical analysis

Patient characteristics were summarized using descriptive statistics including means (standard deviations) and median (interquartile ranges) for continuous variables and frequencies (percentages) for categorical variables. Pearson correlation was used to determine the correlation between BMI and modified CCI. The primary outcome of this study was overall survival, which was calculated from the date of ICI initiation until date of death from any cause or censored at loss to follow-up. Natural Cubic Spline was used to examine the potential non-linear association between BMI and overall survival with BMI = 30 chosen as the reference point. We have merged the groups of patients with normal weight (BMI 18.5-24.9) and overweight (BMI 25.0-29.9) into one category, and similarly combined Obese Class I (BMI 30.0-34.9) with Obese Class II (BMI 35.0-39.9) into another, based on our analysis using the natural cubic spline method. Cox proportional hazard models were first used to assess univariate associations between potential predictors for OS. After assessing collinearity, variables with a p-value < 0.05 (excluding BMI) were then entered into multivariable models for OS. Variables were removed sequentially from the multivariable models via backward selection. The associations of BMI with OS were modeled by Cox proportional hazards regression, controlling for the predictors retained after backward selection (modified CCI, line of therapy, and ECOG) and for cancer type, which was included based on clinical relevance. Adjusted hazard ratios (AHRs) and 95% CIs were displayed using forest plots. Patient characteristics were compared between males and females using two-sample t-test or Kruskal-Wallis test for continuous variables and chi square test for categorical variables. Heterogeneity by sex was evaluated by inserting an interaction term in the model (BMI*Sex). Survival curves were plotted using the Kaplan-Meier method and log-rank p-value was reported. R statistical software (version 4.2.0) was used to plot natural cubic splines and SAS (9.4) was used for the rest of the analysis.

## 3. Results

In our study, a total of 688 patients with metastatic cancers treated with first- or second-line ICIs were included. The mean BMI was 28.8 with standard deviation (SD) of 7.1. The median BMI was 28.0 with interquartile range (IQR) of 23.9 and 32.4. The study cohort included 285 (41%) females and 403 (59%) males, while 649 (94%) were white and 31 (5%) were black. The mean and median ages were 62 (with SD of 12.6) years and 62 (with IQR 54.3 and 70.3) years, respectively. A total of 360 patients (52%) received ICIs as first-line therapy and 328 (48%) received ICIs as second-line therapy. The majority of patients (569, 83%) had Eastern Cooperative Oncology Group performance status (ECOG) of 0 or 1 (Table 1).

**Figure 1:**
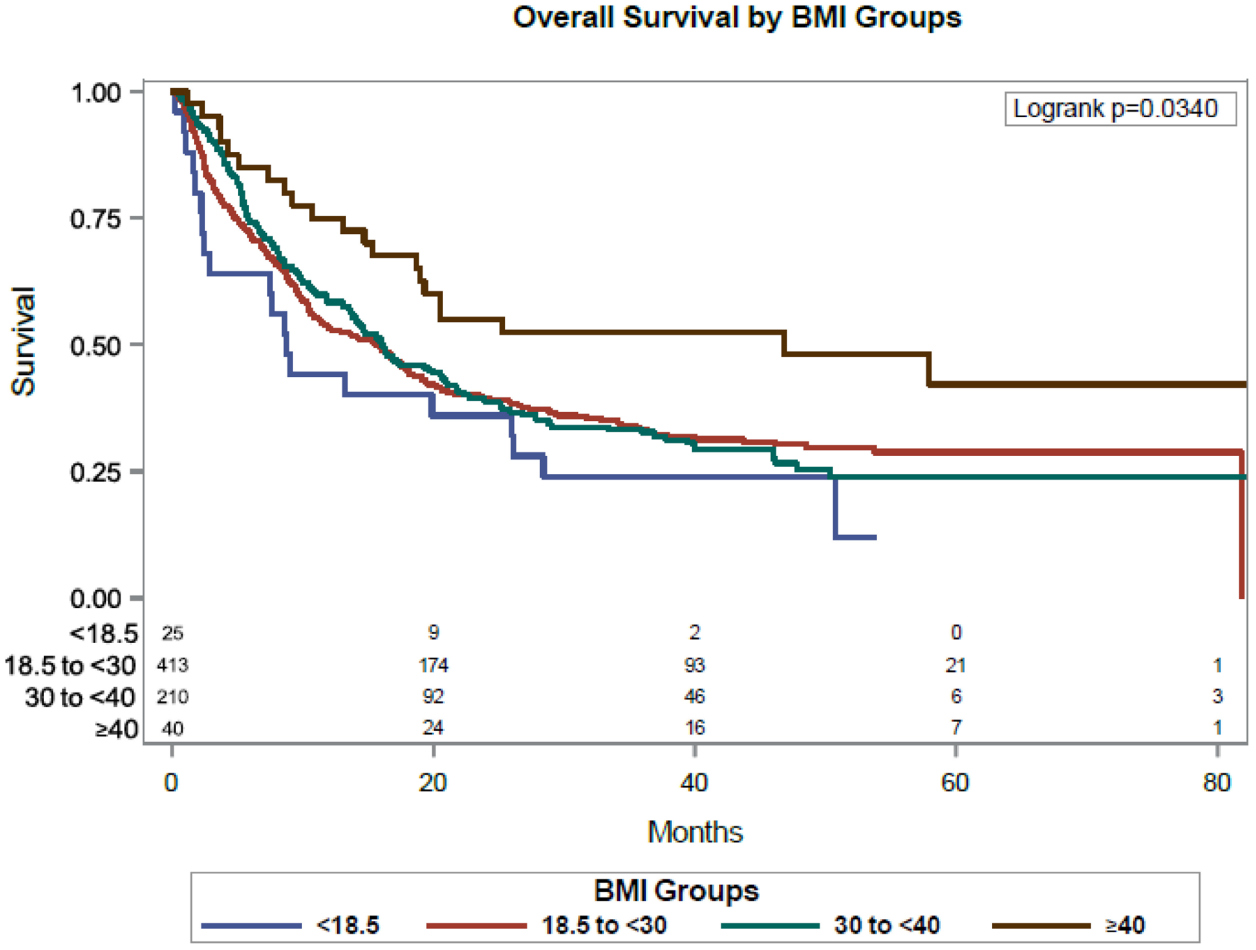
Kaplan-Meier analysis of overall survival Categorized by BMI. This analysis illustrates that patients with extreme obesity (BMI ≥ 40) exhibit the longest overall survival. BMI denotes body mass index.

In this cohort, higher BMI was associated with longer OS. In univariate analysis, for every 10 unit increased in BMI, the hazard ratio (HR) of death decreased by 17% [HR = 0.83, 95% confidence interval (CI) 0.73-0.95, p = 0.007]. In natural cubic spline analysis, BMI < 20 was associated with accelerated increase in HR for death, whereas BMI ≥ 40 was associated with greater decline in HR (Supplemental Figure 1). In analyses using categorical BMI, compared to patients with BMI 18.5 to < 30, patients with morbid obesity (BMI ≥ 40) had a significantly decreased risk in death (HR 0.58, 95% CI 0.37-0.90) (Figure 1).

To assess for underlying comorbidities, we used modified CCI by excluding points assigned to cancer. The median modified CCI was 3, and IQR 1 to 4. In univariate analysis, higher modified CCI at baseline was associated with increased mortality, HR 1.12 with 95% CI 1.08-1.17 for every point increase in modified CCI. There was no significant correlation between BMI and modified CCI. Pearson correlation coefficient was 0.011 and 2-tailed p-value was 0.77. In analysis of individual CCI components, significant associations were found between BMI groups and chronic obstructive pulmonary disease (p-value = 0.0438) and diabetes mellitus (p-value < 0.001).

In addition to BMI and modified CCI index, cancer type, line of therapy, ECOG performance status, smoking status, diabetes mellitus, type of immunotherapy was associated with overall survival (Table 1).

**Figure 2:**
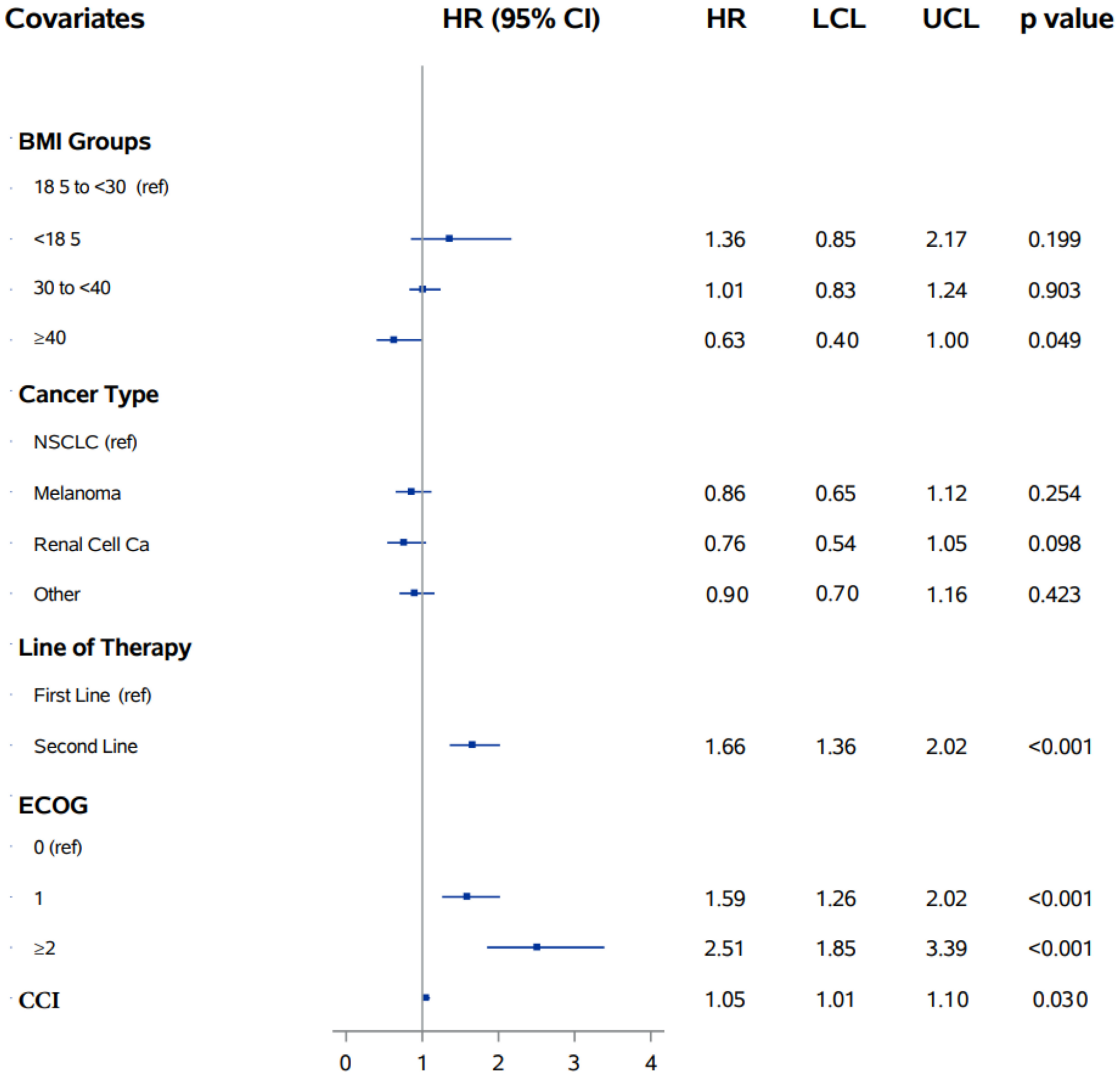
Multivariate analysis of BMI, cancer types, line of therapy, ECOG performance status, and modified CCI. This analysis reveals that a BMI ≥ 40, line of therapy, ECOG performance status, and the modified CCI are significant predictors of overall survival. BMI denotes body mass index; CCI, Charlson Comorbidity Index; ECOG, Eastern Cooperative Oncology Group; NSCLC, non-small cell lung cancer.

In multivariate analysis involving BMI group, modified CCI, cancer type, line of therapy, and ECOG performance status, BMI ≥ 40, line of therapy, ECOG, modified CCI index remained as significant predictor of death with p-value < 0.05 (Figure 2).

### Sex Difference

We observed there is an association between BMI≥40 and overall survival in the male population, but not in the female population. In unadjusted model, for men, patients with BMI ≥40 had 65% lower hazard of death than those with BMI 18.5 to <30 (HR = 0.35, 95% CI 0.18-0.69). For women, there was no significant difference between BMI ≥40 and BMI 18.5 to <30 group. The interaction term for BMI ≥40 and sex was statistically significant (p = 0.016), indicating effect modification by sex for extreme obesity.

In multivariate analysis, after controlling for cancer type, ECOG performance status, line of therapy, and modified CCI, in males, BMI ≥40 remained a statistically significant predictor of OS (HR=0.44, 95% CI 0.22-0.91). Whereas for females, HR was 0.89 (0.49-1.63). Due to relatively low numbers of patients with BMI ≥40, the difference in HR between males and females was not significant, with the p-value for interaction term (sex*BMI) at 0.144 (Supplemental Table 2).

## 4. Discussion

The relationship between obesity, oncogenesis and cancer treatment outcome is complex. We hypothesized that immune dysfunction seen in obesity-related cancers contributed to longer overall survival for patients treated with ICI. In this study, we found that higher BMI was associated with improved overall survival in cancer patients treated with ICI. These findings are consistent with earlier studies linking higher BMI with survival 20, 21. In addition, the greatest survival benefit was seen in patients with extreme obesity (BMI ≥40). Interestingly, in subgroup analysis, the association of BMI with longer survival was seen only in male patients and not in females.

To better assess the association between BMI and overall survival, we compiled a modified CCI to assess any potential underlying medical comorbidity that might have influenced treatment outcomes. The modified CCI in our study was strongly predictive of overall survival as previously reported 32. However, we did not find a statistically significant correlation between increased BMI and modified CCI in our patients. The positive association between BMI and modified CCI were seen in multiple large population studies, and often not significant in smaller cohorts 33, 34. In multivariate analysis after adjusting for ECOG performance status, cancer type, line of therapy, and modified CCI, BMI remained a statistically significant prognostic factor for overall survival.

Interestingly, a sex-biased association exists in our study between overall survival and increased BMI. In bivariate analysis, significant longer overall survival was observed only in male patients with extreme obesity (BMI ≥40). Furthermore, the difference in hazard ratios for death between males (HR 0.35, 95% CI 0.18-0.69) and females (HR 1.07, 95% CI 0.59-1.94) was substantial, with p=0.016 for the interaction term. In the multivariate analysis, extreme obesity remained a significant predictor of overall survival (OS) in males, but not in females, after adjustments for performance status, cancer type, line of therapy, and modified CCI. However, it is noteworthy that the difference in hazard ratios between males (HR 0.44, 95% CI 0.22-0.91) and females (HR 0.89, 95% CI 0.49-1.63) was not statistically significant, as indicated by a non-significant interaction between sex and BMI (p = 0.144), after adjusting for multiple potential confounders in our relatively small patient population with extreme obesity. To validate our findings, future studies with larger sample sizes are needed.

We hypothesize that the difference in male and female patients may be due to the differences in body composition between sex in our cohort. With the same BMI, males typically have higher visceral fat 35, 36. Lower circulating androgen levels are associated with increased intra-abdominal fat in males 37. In conjunction with recent published data, our clinical data lends support to androgen mediated T-cell exhaustion 26-28. However, we do acknowledge our cohort had significantly fewer females, and only a low percentage of patients had extremely elevated BMI. Therefore, these analyses should be repeated in larger cohorts.

In addition, the positive association between high BMI and overall survival could contributed by the larger energy reserve in patients with obesity, which may offer greater ability to tolerate cancer treatment 38, 39. It is well known that cancer associated cachexia is associated with poor outcome 40, 41. BMI also reflects underlying body composition and affects pharmacokinetic/pharmacodynamic dosing of ICI with chemotherapies 17, 42, 43. The effects of obesity on overall survival were beyond the scope of this project.

Taken together, although higher BMI was associated with improved overall survival, the effect size was modest and largely driven by patients with extreme obesity (BMI ≥40), indicating a nonlinear relationship. The nonlinear pattern suggests that the observed survival benefit may reflect underlying obesity-associated immune and metabolic states present in extreme obesity, rather than incremental differences in BMI alone. Importantly, these findings should not be interpreted as a recommendation for weight gain, as BMI likely reflects underlying host factors that influence response to immune checkpoint inhibition.

In our study, only the minority of patients (12%) had known PD-L1 status. Therefore, this variable was not included in the analysis due to the high level of missingness, which limits our ability to fully account for its potential impact on the overall survival analysis. However, existing clinical evidence regarding a direct association between BMI or obesity and tumor PD-L1 expression is inconsistent and appears to be cancer-specific and sex-dependent 44-46. Notably, prior studies have shown that obesity or higher BMI may be independently associated with improved survival among patients with NSCLC receiving immunotherapy, including those with high PD-L1 expression 23, 47. Larger, cancer-specific and sex-stratified cohorts are needed to further elucidate the relationship between obesity, PD-L1 expression, and immunotherapy outcomes.

This study has demonstrated the association between increased survival and high BMI after adjusting for medical comorbidities. There were several limitations in our study. First, this was a retrospective study and we could not establish any causal relationship. Second, although readily available, BMI could not measure body composition directly and we could not measure the distribution of adiposity 48. Third, we were unable to effectively analyze ICI dosing and combination therapy with other cytotoxic chemotherapies due to the heterogeneity of our cohort. Finally, the lower number of female patients in our cohort could contribute to the lack of association between BMI and overall survival in females. Future studies consisting of a larger, more homogeneous cancer cohort are needed to further delineate some of these relationships.

## 5. Conclusion

Obesity was associated with increased overall survival for cancer patients who have received immune checkpoint inhibitors, independent of underlying medical comorbidities and performance status. Furthermore, the survival benefit related to obesity may vary depending on the patient's sex.

## Supplementary Material

Supplementary figures and tables.

## Figures and Tables

**Table 1:** Patient demographic and clinical characteristics with corresponding univariate hazard ratios (HRs) for overall survival. Cox proportional hazards models were used to examine univariate associations between risk factors and overall survival.

	Mean (SD)	HR (95% CI)	p-value
Age	61.9 (12.6)	1.02 (1.01-1.02)	**< 0.001**
BMI	28.8 (7.1)	0.83* (0.73-0.95)	**0.007**
	Median (IQR)		
CCI	3 1, 4	1.12 (1.08-1.17)	**< 0.001**
	N (percent)		
BMI Groups			**0.038**
18.5 to <30	413 (60%)	Reference	
<18.5	25 (3.6%)	1.40 (0.89-2.20)	
30 to <40	210 (30.5%)	1.00 (0.82-1.21)	
≥40	40 (5.8%)	0.58 (0.37, 0.90)	
Cancer Type			**< 0.001**
NSCLC	157 (22.8%)	Reference	
Melanoma	277 (40.3%)	0.52 (0.41-0.66)	
Renal Cell Ca	67 (9.7%)	0.81 (0.58-1.12)	
Other	187 (27.2%)	0.81 (0.64-1.03)	
Line of Therapy			**< 0.001**
First Line	360 (52.3%)	Reference	
Second Line	328 (47.7%)	1.98 (1.65-2.38)	
ECOG			**< 0.001**
0	286 (41.6%)	Reference	
1	283 (41.1%)	1.91 (1.55-2.34)	
≥2	107 (15.6%)	3.18 (2.46-4.11)	
Unknown	12 (1.7%)		
Race			0.426
White	649 (94.3%)	Reference	
Black	31 (4.5%)	1.30 (0.86-1.98)	
Other	8 (1.2%)	0.84 (0.35-2.03)	
Sex			0.673
Female	285 (41.4%)	Reference	
Male	403 (58.6%)	0.96 (0.80-1.15)	
Smoking status			**0.007**
No	273 (39.7%)	Ref	
Yes	415 (60.3%)	1.29 (1.07, 1.56)	
Diabetes status			**0.012**
No	548 (79.7%)	Ref	
Yes	140 (20.3%)	1.32 (1.06, 1.63)	
Immunotherapy			**0.006**
Anti-PD-1	412 (59.9%)	Reference	
Anti-PD-L1	26 (3.8%)	1.73 (1.12-2.67)	
Anti-CTLA-4	156 (22.7%)	0.79 (0.63-0.99)	
Anti-PD1+CTLA-4	68 (9.9%)	0.89 (0.65-1.20)	
Other	26 (3.8%)	0.66 (0.40-1.09)	

Significant p-values < 0.05 are bolded. *Hazard ratio is reported per 10-unit increase in BMI. BMI denotes body mass index; CCI, modified Charlson Comorbidity Index; CTLA-4, cytotoxic T-lymphocyte antigen-4; ECOG, Eastern Cooperative Oncology Group performance status; IQR, interquartile range; SD, standard deviation; PD-1, programmed cell death protein 1; PD-L1, programmed death-ligand 1.

## Abbreviations

BMI: body mass index
CCI: Charlson Comorbidity Index
CI: confidence interval
ECOG: Eastern Cooperative Oncology Group
HR: hazard ratio
ICI: immune checkpoint inhibitor
IL: interleukin
IQR: interquartile range
NSCLC: non-small cell lung cancer
OS: overall survival
RCC: renal cell carcinoma
STAT3: signal transducer and activator of transcription 3
PD-1: programmed cell death protein 1
PD-L1: programmed cell death-ligand 1
WHO: World Health Organization
