# Supplementary material for: Association between obesity, sex, medical comorbidities, and survival in cancer patients treated with immune checkpoint inhibitors
Source: J Cancer. 2026 Jun 10;17(6):1117–24. doi: 10.7150/jca.130032 (PMC13280536; doi:10.7150/jca.130032)
Supplement: Supplementary file 1 — Supplementary figures and tables. [file jcav17p1117s1.pdf]

Supplemental:

Supplemental Table 1: Modified Charlson Comorbidity Index

Comorbidity	Severity	Points
Age (Years)	<50	0
	50-59	1
	60-60	2
	70-79	3
	≥80	4
Myocardial Infarction	No	0
	Yes	1
Congestive Heart Failure	No	0
	Yes	1
Peripheral Vascular Disease	No	0
	Yes	1
Cerebral Vascular Accident or Transient Ischemic Attacks	No	0
	Yes	1
Dementia	No	0
	Yes	1
Chronic Obstructive Pulmonary Disease	No	0
	Yes	1
Connected Tissue Disease	No	0
	Yes	1
Peptic Ulcer Disease	No	0
	Yes	1
Liver Disease	None	0
	Mild	1
	Moderate to Severe	3
Diabetes Mellitus	None or Diet-Controlled	0
	Uncomplicated	1
	End-organ damage	2

Hemiplegia	No	0
	Yes	2
Chronic Kidney Disease	Mild	0
	Moderate to Severe	2
Acquired Immunodeficiency Syndrome	No	0
	Yes	6

Supplemental Table 1. Medical conditions and their corresponding points for the modified Charlson Comorbidity Index (CCI) utilized in this Study

Supplemental Table 2: Sex-Specific Impact of BMI on Overall Survival

BMI Groups	Sex	Unadjusted model		Adjusted model	
		HR (95% CI)	Interaction *P* value	AHR (95% CI)	Interaction *P* value
<18.5 vs 18.5 to <30	Female	1.36 (0.73, 2.53)	0.779	1.42 (0.76-2.65)	0.967
30 to <40 vs18.5 to <30	Female	1.22 (0.91, 1.65)	0.081	1.16 (0.82-1.51)	0.437
≥40 vs 18.5 to <30	Female	1.07 (0.59, 1.94)	0.016	0.89 (0.49, 1.63)	0.144
<18.5 vs 18.5 to <30	Male	1.55 (0.79, 3.03)		1.39 (0.68, 2.84)	
30 to <40 vs18.5 to <30	Male	0.86 (0.66, 1.12)		0.95 (0.72, 1.25)	
≥40 vs 18.5 to <30	Male	0.35 (0.18, 0.69)		0.44 (0.22, 0.91)	
BMI (continuous , per 10-unit increase)	Female	1.03 (0.86, 1.24)	0.003	0.96 (0.80, 1.15)	0.154
	Male	0.69 (0.57, 0.83)		0.79 (0.65, 0.96)	

Supplemental Table 2. Sex-Specific Impact of BMI on Overall Survival. Interaction p-values for categorical BMI represent sex-stratified estimates within each BMI category and are not derived from a single global BMI×sex interaction term. In the unadjusted model, extreme obesity (BMI ≥40) significantly predicts longer overall survival exclusively in males. Additionally, there is a significant difference in hazard ratio (HR) for death between males and females, with a p-value of 0.016 for the interaction term (BMI*Sex). In the adjusted model, which accounts for variables such as cancer type, ECOG performance status, line of therapy, and modified CCI, a BMI ≥40 continues to be a significant predictor of overall survival in males, but not in females. Notably, in this adjusted model, the difference in HR for death between sexes is not significant, with a p-value of 0.144 for

the interaction term (BMI*Sex). BMI denotes BMI; CCI, modified Charlson Comorbidity Index; ECOG, Eastern Cooperative Oncology Group; HR, hazard ratio.

Supplemental Figure 1: Analysis of the Nonlinear Relationship Between Body Mass Index (BMI) and Overall Survival Using Natural Cubic Splines

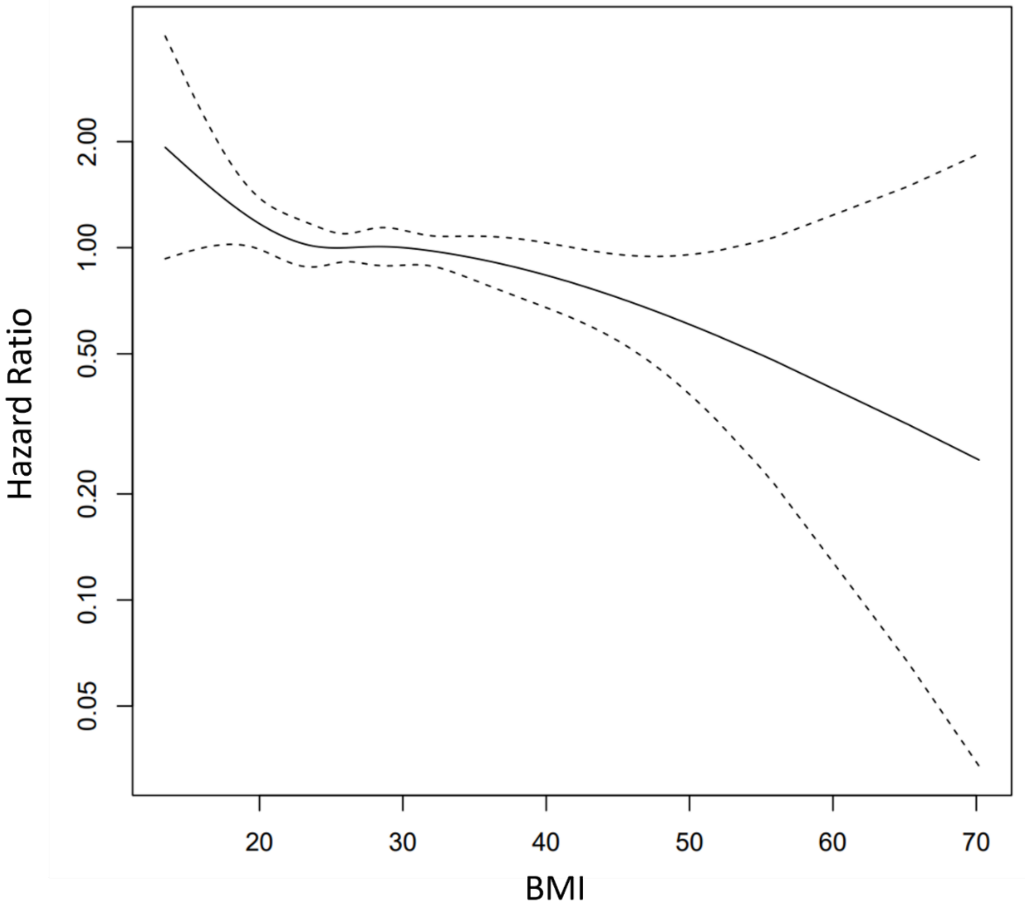

Supplemental Figure 1. Natural Cubic Spline Analysis of the Nonlinear Association Between BMI and Overall Survival. The spline model illustrates that the HR for death decreases steadily with an increase in BMI, maintaining a relatively constant rate between a BMI of 20 and 40. Notably, there is a marked increase in HR for death when BMI is below 20, and a significant decrease in HR as BMI exceeds 40, indicating a nonlinear and differential impact of BMI extremes on survival outcomes. BMI denotes body mass index; HR, Hazard Ratio.
